# Zika Virus Vector Competency of Mosquitoes, Gulf Coast, United States 

**DOI:** 10.3201/eid2303.161636

**Published:** 2017-03

**Authors:** Charles E. Hart, Christopher M. Roundy, Sasha R. Azar, Jing H. Huang, Ruimei Yun, Erin Reynolds, Grace Leal, Martin R. Nava, Jeremy Vela, Pamela M. Stark, Mustapha Debboun, Shannan Rossi, Nikos Vasilakis, Saravanan Thangamani, Scott C. Weaver

**Affiliations:** University of Texas Medical Branch, Galveston, Texas, USA (C.E. Hart, C.M. Roundy, S.R. Azar, J.H. Huang, R. Yun, E. Reynolds, G. Leal, S. Rossi, N. Vasilakis, S. Thangamani, S.C. Weaver);; Harris County Public Health, Houston, Texas, USA (M.R. Nava, J. Vela, P.M. Stark, M. Debboun)

**Keywords:** Zika, Zika virus, vector, competence, *Culex quinquefasciatus*, *Aedes taeniorhynchus*, mosquitoes, viruses, vector-borne infections, Gulf Coast, United States

## Abstract

Zika virus has recently spread throughout the Americas. Although *Aedes aegypti* mosquitoes are considered the primary vector, *Culex quinquefasciatus* and mosquitoes of other species may also be vectors. We tested *Cx. quinquefasciatus* and *Ae. taeniorhynchus* mosquitoes from the US Gulf Coast; both were refractory to infection and incapable of transmission.

Although most human Zika virus infections produce no symptoms or only mild febrile illness, the association with microcephaly and other severe congenital defects has caused a public health crisis since the virus arrived in the Americas. Part of the concern is local, mosquitoborne transmission in the United States (*1*). *Aedes* (*Stegomyia*) *aegypti* mosquitoes are believed to be the primary urban Zika virus vectors, according to laboratory transmission studies (*2*) including vertical (*3*) and natural Zika virus infections detected in Malaysia (*4*) and during a 2015 Mexico outbreak (*5*). This highly anthropophilic mosquito occurs nearly throughout the tropics and subtropics, including the southern United States. However, in many tropical and subtropical regions, the most abundant urban mosquito is *Culex quinquefasciatus*. One experimental study found that *Cx. quinquefasciatus* mosquitoes from China are capable of Zika virus transmission (*6*), and others found that mosquitoes of this species and the closely related *Cx. pipiens* are refractory to Zika virus infection (*2*). Surveillance during an outbreak in Mexico also found no evidence of natural *Cx. quinquefasciatus* mosquito infection in regions of active transmission (*5*).

One US region at highest risk for Zika virus circulation is the Gulf of Mexico coast (Gulf Coast), especially Houston, Texas, which is a major hub for air transportation and has large populations of *Ae. aegypti* mosquitoes. Evidence of past dengue virus circulation (*7*) also suggests permissive conditions for Zika virus transmission. However, the most abundant mosquitoes immediately along the Gulf Coast are typically salt marsh species, such as *Ae.* (*Ochlerotatus*) *taeniorhynchus*, a competent vector for arboviruses, including Venezuelan equine encephalitis virus. Mosquitoes of this species are widely distributed in North, Central, and South America, and their mammalophilic feeding behavior could enable transmission of arboviruses among humans (*8*).

To determine if *Cx. quinquefasciatus* mosquitoes are capable of Zika virus transmission, we fed cohorts of 50 mosquitoes (colonized and reared in an insectary) artificial blood meals containing 10^4^ to 10^6^ focus-forming units (FFU)/mL of virus prepared in Vero cell cultures. Fully engorged mosquitoes were incubated at 27°C and 80% humidity and provided aqueous sucrose ad libitum. Multiple Zika virus strains were fed to the mosquito cohorts: FSS13025 (2010 Cambodia, closely related to strains from the Americas), DAKAR41525 (1985 Senegal), and MEX1–7 (isolated from a 2015 outbreak in Mexico) (*5*). On days 3, 7, and 14 after mosquito feeding, we homogenized bodies and legs from 20 mosquitoes and tested them for Zika virus by focus-forming assay; on days 7 and 14, we also tested saliva.

Because natural blood meals from viremic animals are typically more infectious for mosquitoes than are artificial meals (*9*), we allowed 3 groups of *Cx. quinquefasciatus* mosquitoes to feed on FSS13025-infected type I interferon-receptor knockout A129 mice on postinfection days 1, 2, and 3, corresponding to viremia titers of 10^4^, 10^7^, and 10^6^ FFU/mL, respectively, as determined by back-titration of mouse blood collected immediately after feeding. A separate mouse was used for each infection. On days 3, 7, and 14, we subjected mosquito bodies, legs, and saliva to focus-forming assay. All samples were also negative for Zika virus (Table).

**Table:** Potential mosquito vectors of southern United States that showed no infection, dissemination, or transmission of Zika virus*

Virus strain	Mosquito species/strain	Blood meal	Dose, log_10_ FFU/mL	No./time point	Days tested after feeding
MEX 1–44 (Mexico 2015)	*Culex quinquefasciatus* (colonized)	Artificial	6	20	10, 17
	*Aedes taeniorhynchus* (colonized)	Artificial	6	20	10, 17
DAK AR 41525 (Senegal 1985)	*Cx. quinquefasciatus* (colonized)	Artificial	4, 5, 6	20	3, 7, 14
FSS 13025 (Cambodia 2010)	*Cx. quinquefasciatus* (colonized)	Artificial	4, 5, 6	20	3, 7, 14
	*Cx. quinquefasciatus* (Houston F2)	Murine	4, 6, 7	5	3, 7, 14
MEX 1–7 (Mexico 2015)	*Cx. quinquefasciatus* (colonized)	Artificial	4, 5, 6	20	3, 7, 14
	*Cx. quinquefasciatus* (Houston F2)	Murine	6	26	14
PRABC59 (Puerto Rico 2015)	*Cx. quinquefasciatus* (Houston F2)	Murine	7	21	14

*Infection, dissemination, and transmission rates were all 0. FFU, focus-forming units.

To preclude the possibility that laboratory colonization diminished *Cx. quinquefasciatus* mosquito competence for Zika virus transmission, we collected F2 mosquitos from the Houston area and also allowed them to feed on A129 mice 2 days after infection with FSS13025, MEX1–7, or Puerto Rico strain PRVABC59, with viremia titers of 10^7^, 10^6^, and 10^7^ FFU/mL, respectively. None of the bodies, legs, and saliva samples collected 14 days after feeding were positive for Zika virus.

*Ae. taeniorhynchus* mosquitoes were also tested for Zika virus transmission competence. Colonized mosquitoes were fed artificial blood meals containing 10^6^ FFU/mL Zika virus (strain MEX1–44), and on days 10 (n = 20) and 17 (n = 20), salivary glands, legs, and midguts were dissected and screened for virus by infectious assays (*3*). None of the mosquito samples was positive for Zika virus (Table).

Our results concur with those of others showing the inability of Zika virus to infect *Culex* spp. mosquitoes (*2*). We also found that *Ae. taeniorhynchus* mosquitoes from the Gulf Coast are refractory to Zika virus infection. The Zika virus strains and actual stocks used for our experiments were infectious for *Ae. aegypti* mosquitoes in other experiments (C. Roundy et al., unpub. data), indicating that our negative findings for *Cx. quinquefasciatus* and *Ae. taeniorhynchus* mosquitoes represent truly refractory phenotypes. These results, along with findings from an outbreak in southern Mexico (*5*), support the conclusion that *Ae. aegypti* mosquitoes are the primary urban Zika virus vectors. However, regional variation in competence could be reflected in the study from China that shows Zika virus presence in saliva after experimental infection (*6*). Additional research is needed to understand whether this putative geographic variation reflects mosquito genetics or other intrinsic factors, such as microbiome or microvirome populations within this species. Because some studies indicate that *Cx. quinquefasciatus* mosquitoes are more ornithophilic than mammalophilic, including in parts of China (*10*), their feeding habits in regions where they are transmission competent require evaluation to assess their true capacity as vectors.
